# Antibiotic resistant *Cutibacterium acnes* among acne patients in Jordan: a cross sectional study

**DOI:** 10.1186/s12895-020-00108-9

**Published:** 2020-11-17

**Authors:** Eman Alkhawaja, Saleem Hammadi, Medhat Abdelmalek, Naser Mahasneh, Bayan Alkhawaja, Suzanne M. Abdelmalek

**Affiliations:** 1 Department of Pharmacology and Biomedical Sciences, Faculty of Pharmacy, Petra University, Amman, Jordan; 2 Consultant Dermatologist, private clinic, Amman, Jordan; 3 Department of Dermatology, Faculty of Medicine, Jordan University, Amman, Jordan; 4 Department of Pharmaceutical Medicinal Chemistry & Pharmacognosy, Faculty of Pharmacy, Petra University, Amman, Jordan

**Keywords:** *Cutibacterium acnes*, Acne, Antibiotic resistance patterns, Misuse of antibiotics, *MDR*

## Abstract

**Background:**

Antibiotics have been used for decades against *Cutibacterium acnes* (previously known as *Propionibacterium acnes; C. acnes)*. Alarmingly, antibiotic resistance to this bacterium has become a worldwide problem in recent years. No studies are available on the antibiotic susceptibility patterns of *C. acnes* among Jordanian acne patients and how that is influenced by antibiotic use.

This study aims to assess antibiotic resistance patterns of *C. acnes* clinical isolates and neighboring Gram-positive normal flora of the skin obtained from acne patients attending dermatology clinics in Amman –Jordan appraising the role of antibiotic consumption.

**Methods:**

This is a cross-sectional study of acne patients presenting to selected dermatology outpatient clinics over a 6-month study period. Swabs obtained from inflamed lesions were cultured aerobically and anaerobically. Isolates were identified and screened for antibiotic susceptibility. In addition, all patients were asked to fill in a questionnaire that included questions about the history of antibiotic treatment.

**Results:**

*C. acnes* was isolated from lesions of 100 patients out of 115 participants included in this study. 73% of the isolates were resistant to erythromycin and 59% to clindamycin 37% to doxycycline, 36% to tetracycline, 31% to trimethoprim / sulfamethoxazole, 15% to levofloxacin, and 3% to minocycline. Multi drug resistance (MDR) in *C. acnes* isolates as well as *Staphylococcus aureus* (*S. aureus*) and *Staphylococcus epidermidis (S. epidermidis*) with a similar pattern of resistance were detected from the same patient in most cases. A pattern of higher resistance towards variable antibiotic was observed in patients previously treated with antibiotics for acne management.

**Conclusions:**

The findings of this study demonstrate the distribution of antibiotic resistance of *C. acnes* towards used antibiotics and emphasizes the influence of antibiotic consumption on development of antibiotic resistance. The similar pattern of resistance between skin bacteria tested in this study highlights the genetic transfer of resistance between skin commensals including *S. aureus* and *S. epidermidis* hence promoting its circulation in the community.

## Background

Acne is the most common dermatologic disorder in humans. It affects mostly adolescents (80%) at the age of puberty when hormonal changes begin and impacts both genders similarly. Resolution of acne begins as patients reach their twenties, nevertheless; acne persists in 54% of adult women and 40% of adult men [1, 2]. It follows the distribution of the sebaceous glands in the body hence it forms on the face, along the jaw line (which is a common location in adults), neck, chest, and back [3, 4]. The disease manifests as white comedons, black heads, moderate acne, or inflamed acne. The pathogenesis of acne is complicated; it develops as a result of hormonal imbalance leading to overproduction of sebum accompanied by follicular duct hyper-keratinisation. The accumulation of dead skin and fatty acids pave the way for *Cutibacterium acnes* (*C. acnes)* follicular enhanced colonization and the subsequent release of inflammatory mediators [5, 6]. *C. acnes,* commonly known as the cause of acne vulgaris, is typically an aerotolerant anaerobic, Gram-positive bacillus that colonizes the pilosebaceous follicle, derives energy from hydrolyzing triglycerides of the sebum, and secretes fatty acid byproducts, mainly propionic acid. These byproducts can irritate the follicular wall and induce inflammation through neutrophilic chemotaxis [7]. *C. acnes* promotes the abnormal proliferation and differentiation of keratinocytes as well as further sebum production. In addition, *C. acnes* activate the innate immune system through the overexpression of toll-like receptors, protease-activated receptors and matrix metalloproteinase by keratinocytes [8].

As a result of the complex pathogenicity of this condition, management is likewise diverse, including medications that control hormones, bacterial growth and the inflammatory response resulting from chemotactic factors produced by these bacteria. Control of bacterial growth is achieved by the use of antibiotics. Oral antibiotics are the standard treatment for moderate acne or for cases in which topical combinations were not tolerated or were ineffective [9]. Systemic erythromycin and different generations of tetracyclines are known to be effective in the management of inflammatory acne [10]. Vitamin A derivatives in the form of topical retinoids normalize the life cycle of follicles by preventing or reducing hyperkeritinization. Oral retinoid- (Isotretinoin) on the other hand, shrink the sebaceous glands, reduce sebum, cellular debris and metabolic byproducts from the surrounding skin tissue, which in turn reduces the amount of comedones produced [5, 11].

Given that both topical and oral antibiotics against *C. acnes* have been used for decades, *C. acnes* resistance to used antibiotics, such as erythromycin and clindamycin has been detected with high prevalence in Mediterranean countries mainly due to antibiotic abuse. For instance, researchers from Spain reported 91 and 92.4% resistance respectively for both antibiotics. The rates of resistance in Greece were and 75.3% for both antibiotics and they were 59.5% in Italy [12]. The increasing number of *C. acnes* drug-resistant strains has provoked global concerns over the decreasing number of antibiotics that can be used to treat this common condition [13].

Antibiotic resistance in *C. acnes* can spread to other commensal bacteria colonizing the skin and the follicles such as *S. epidermidis* through horizontal transfer of resistance genes [14, 15]. Therefore, careless administration of antibiotics would not only result in emerging antibiotic resistant *C. acnes* but also the spread of resistance to other bacterial species [14].

So far, there is no available data regarding antibiotic resistance of *C. acnes* among Jordanians. This study aims to determine the prevalence of antibiotic resistance in *C. acnes* isolates from acne patients attending private and public dermatology clinics in Amman -Jordan.

## Methods

### Patients

115 acne outpatients attending private and public dermatology clinics in Amman, Jordan were included in this study throughout a 6-month study period. Participants were asked to sign a consent form that included the methodology of the research and requested their approval for obtaining a microbiological sample from their inflamed pustules.

### Sample collection

A dermatologist or a trained nurse were asked to obtain the sample after decontaminating the skin and the infected areas with 70% alcohol. Sterile cotton swabs were used to obtain the samples from a lesion. The lesion was opened aseptically in the clinic and swabbed. Swabs were aseptically placed in a Thioglycolate media tube (HI media-India). Another swab was obtained from the adjacent skin to the lesion. Swabs were sent to the lab for further analysis.

### Microbiological isolation and identification

Thioglycolate tubes containing inoculated sample swabs were incubated at 37 °C for 7 days and then sub cultured on blood agar aerobically and anaerobically using OXOID Anaerobic jar containing Anaerogen kit (Oxoid-Germany) for 7 days at 37 °C. Identification of bacterial isolates was performed according to colonial morphology, gram stain and biochemical reactions using RapID ANA п system (REMEL-USA).

### Antibiotic susceptibility testing

Antibiotic susceptibility of isolates was determined by the disc diffusion assay according to the Clinical and Laboratory Standards Institute (CLSI) guidelines with some modifications. Briefly, five colonies were picked from blood agar culture and emulsified in 5 ml of Mueller Hinton Broth MHB (Himedia- India) [12, 16]. A sterile swab was soaked in the suspension and seeded on the surface of Mueller Hinton Agar (MHA). Antibiotic discs 30 μg tetracycline (TE), 30 μg minocycline (MH), 30 μg doxycycline (DO), 15 μg erythromycin (E), 2 μg clindamycin (DA), 1.25/23.75 μg trimethoprim/sulfamethoxazole (SXT), 5 μg Levofloxacin (LEV) (Oxoid (Germany) were then applied on the surface of the agar. Plates were incubated for 7 days in anaerobic conditions for *C. acnes* and overnight for other isolates. At the end of the incubation period, zones of inhibition were measured in millimeters. Experiments were performed in triplicates. The results were interpreted as resistant or sensitive according to the zone diameter interpretive chart (as per NCCLS January 2015) presented by Abdelfattah, Darwish, and Zandi et al. [12, 17, 18]

### History of antibiotic consumption

A questionnaire was distributed to the dermatologist or the trained nurse to collect data from patients. The questionnaire was divided into three sections: first section *demography* which included age, gender and type of clinic whether private or public Second section: *acne classification* whether mild: few to several pustules and papules or moderate: several to many pustules with few nodules or severe: several to many papules and pustules with many nodules. The third section was *history of antibiotics use*: which asked if the patient had been treated with antibiotics during the prior year or ever, whether oral, topical, or combination antibiotics were used, duration of treatment, and the type of antibiotic used.

### Statistical analysis

The data collected were coded and entered into SPSS 17.0 software. Descriptive analysis (frequencies, mean and standard deviation) as well as statistical significance testing was performed using chi-square and t-test tests. The statistical significance was defined as a *P* value ≤0.05.

## Results

### Patients demographics

Samples were obtained from 115 patients visiting public and private dermatology clinics. Eighty-seven patients were females while twenty-eight were males with a female to male ration of 2:1. The mean age of patients was 20.5 ± 4 years and the age of 63% of patients was equal to or greater than 20 years.

The patients experienced various degrees of severity of acne with moderate pustular acne being the predominant amongst them. Percentage of female patients with severe acne was higher than males.

**Fig. 1:**
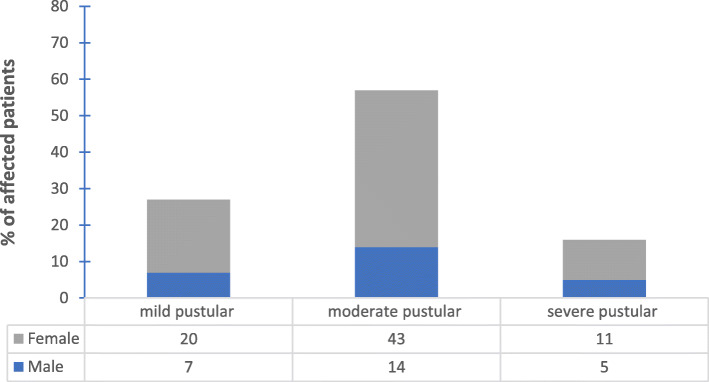
The relation between gender and severity of acne

**Fig. 2:**
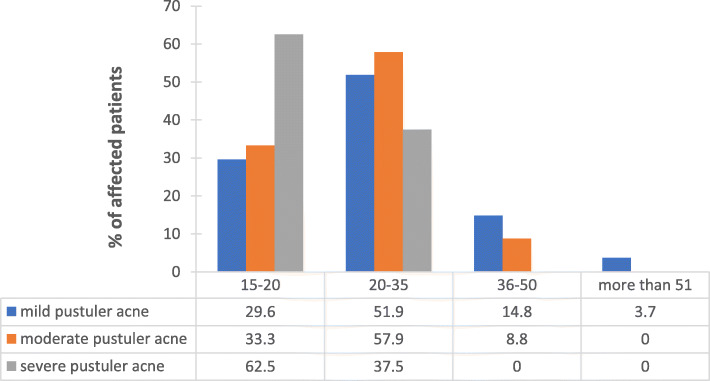
The relation between age of patient and severity of acne condition

*C. acnes* was isolated from 100 patients; 76 females with mean age 20 ± 3.5 years and 24 males with mean age 19 ± 4.9 years. Twenty-seven (27%) patients of those had mild pustular acne, 57 (57%) had moderate pustular acne and 16 (16%) patients had severe pustular acne. The relationship between gender and acne severity was demonstrated in Fig. 1. The severity of acne diminished with age as patients ≥20 years exhibited less severe acne (Fig. 2).

### Microorganisms isolated from acne patients

One hundred and fifteen patients were enrolled in this study. *C. acnes* strains were isolated from 100 (87%) patients; *Staphylococcus epidermidis* strains were also isolated together with *C. acnes* from the skin of 42 (36.5%) patients, while *Staphylococcus aureus* stains were isolated from 49 (42.6) patients. The three stains were isolated from 7 (6.1%) patients (Table 1).

**Table 1:** Distribution of bacterial isolates from patients

Number of samples	Acne lesion	skin	
	***C. acnes***	***S. aureus***	***S. epidermidis***
42	**+**	**–**	**+**
49	**+**	**+**	**–**
7	**+**	**+**	**+**
2	**+**	**–**	**–**
15	**–**	**–**	**–**

### Antimicrobial susceptibility of *C. acnes* isolated from acne patients

*C. acnes* was isolated from lesions of 87% of acne patients. 100% of the isolates showed reduced susceptibility to one or more than one antibiotic.

**Fig. 3:**
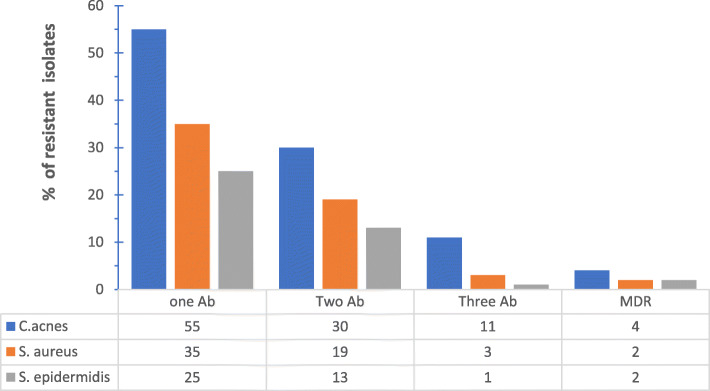
Percentage of resistant isolates to antibiotics. Where Ab: antibiotic, MDR: multi-drug resistance

Clinical isolate strains of *C. acnes* showed variable resistance to the used antibiotics. It ranged from resistance to one antibiotic to multidrug resistance that involved different antibiotics having different mode of actions (Fig. 3). Erythromycin and clindamycin exhibited the highest resistance percentages among tested antibiotics (Table 2).

**Table 2:** Percentage of *C. acnes* isolates resistant to different antibiotics

Antibiotic	Concentration (μg)	Sensitive^**a**^ n(%)	Resistant^**a**^ n(%)
**Doxycycline**	30	(63) 63%	(37) 37%
**Tetracycline**	30	(64) 64%	(36) 36%
**Erythromycin**	15	(27) 27%	(73) 73%
**Clindamycin**	2	(41) 41%	(59) 59%
**Trimethoprim/sulfamethoxazole**	12.5/23.75	(69) 69%	(31) 31%
**Levofloxacin**	5	(85) 85%	(15)15%
**Minocycline**	30	(97) 97%	(3) 3%

^a^Determined according to breakpoints ranges [12, 17, 18].

**Fig. 4:**
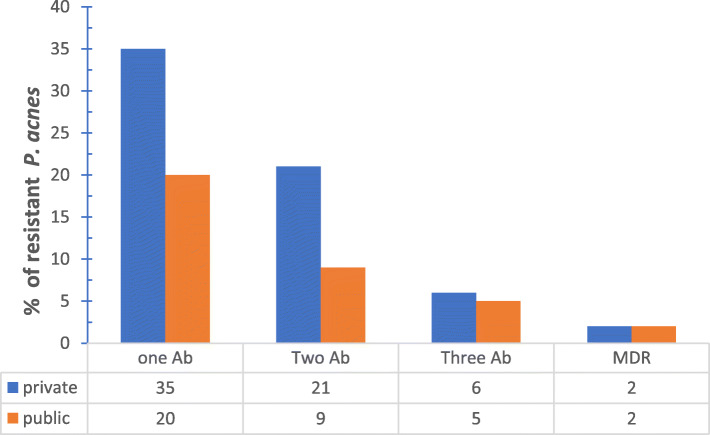
Distribution of single and multiple antibiotic resistant *P. acnes* strains between private and public clinics. Where Ab: antibiotic, MDR: multi-drug resistance

The number of patients carrying antibiotic resistant *C. acnes* and attending private clinics exceeded those attending public clinics, however this difference was not significant and conclusions cannot be drawn from it due to the small sample size and potential confounders (Fig. 4).

### Antibiotics history

**Fig. 5:**
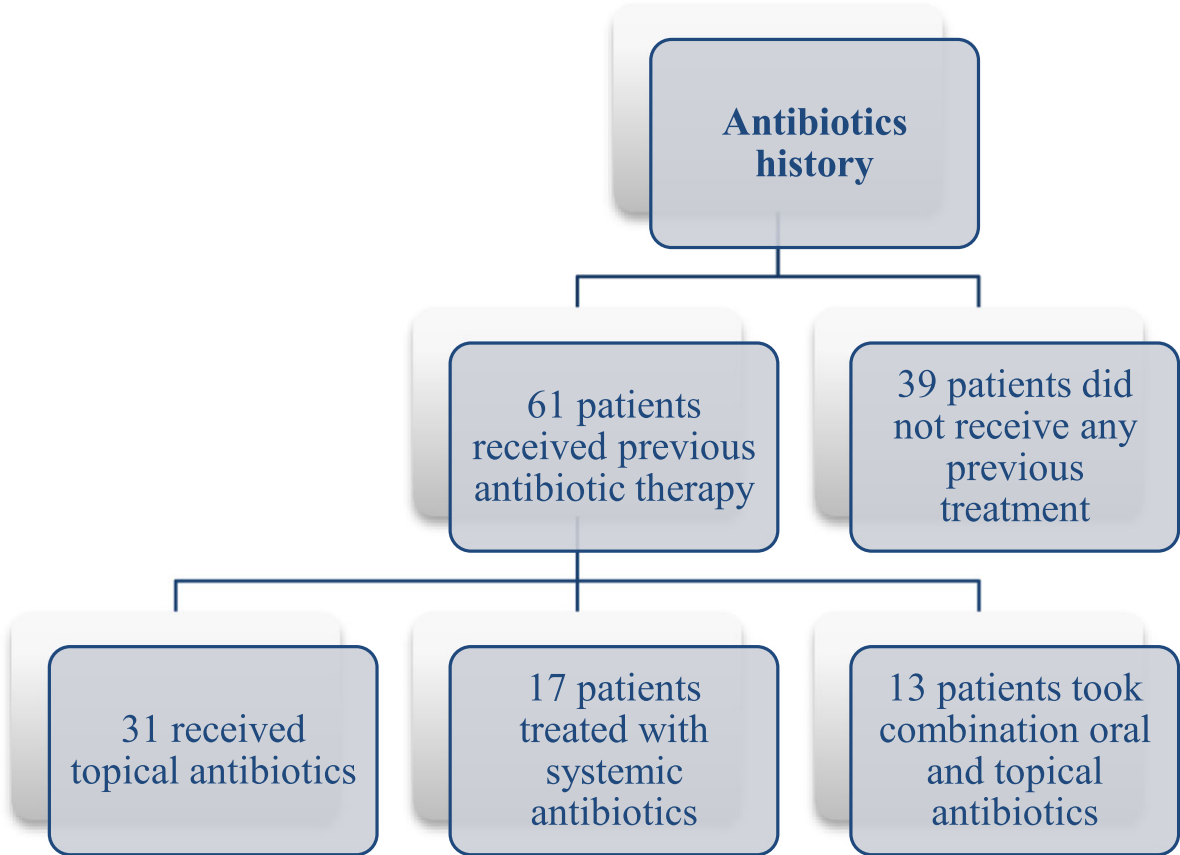
Antibiotic administration history amongst the patients

Regarding previous use of antibiotic therapy for acne treatment, patients were divided into two groups: patients who did not receive prior treatment for acne 39 (39%) and patients who had received prior antibiotic therapy 61 (61%). The with mean treatment duration for patients who had received prior therapy was 6 ± 2 weeks. Thirty-one of those patients (50.8%) had received topical antibiotics (clindamycin (29.1%) and erythromycin (70.9%)), 17 patients (27.9%) were treated with systemic antibiotics (doxycycline) and 13 (21.3%) patients were treated with both oral and topical antibiotics simultaneously (Fig. 5).

The distribution of resistance in C. *acnes* isolates was mainly towards erythromycin, doxycycline and clindamycin which are the most commonly administrated antibiotics for acne management between patients (Table 2). No significant difference was found between the two groups in their resistance profile towards the tested antibiotics yet a higher number of resistant isolates with variable antibiotic resistance was observed in the previously treated group.

### Antimicrobial susceptibility of *S. aureus* and *S. epidermidis* isolated from acne patients

*S. epidermidis* and *S. aureus* were isolated from the skin of 85.5% of patients. The antimicrobial susceptibility patterns from the total sample (91 patients) are shown in (Table 3).

**Table 3:** Antibiotic susceptibilities of isolated *S. aureus* and *S. epidermidis*

Antibiotic	Sensitive (%)^**a**^		Resistant (%)^**a**^		Cross-resistance with ***C. acnes*** (%) ^**a**^	
	***S. aureus***	***S. epidermidis***	***S. aureus***	***S. epidermidis***	***S. aureus***	***S. epidermidis***
**Doxycyclin**	26 (53%)	18 (43%)	23 (47%)	24 (57%)	6 (12%)	4 (10%)
**Tetracyclin**	33 (67%)	22 (52%)	16 (33%)	20 (48%)	5 (10%)	3 (7%)
**Erythromycin**	11 (22%)	4 (10%)	38 (78%)	38 (90%)	11 (22%)	10 (23%)
**clindamycin**	18 (37%)	7 (17%)	31 (63%)	35 (83%)	9 (18%)	8 (19%)
**Trimethoprim / sulfamethoxazole**	23 (47%)	11 (26%)	26 (53%)	31 (74%)	5 (10%)	5 (12%)
**Levofloxacin**	44 (90%)	30 (71%)	5 (10%)	12 (29%)	–	–
**Minocycline**	45 (92%)	32 (76%)	4 (8%)	10 (24%)	–	–

^a^Determined according to breakpoints ranges [12, 17, 18].

Isolates that showed cross-resistance with *C. acnes* were isolated *(data not shown)*. Four isolates showed cross-resistance towards erythromycin between the three studied stains while three isolates showed cross resistance between strains towards clindamycin.

**Fig. 6:**
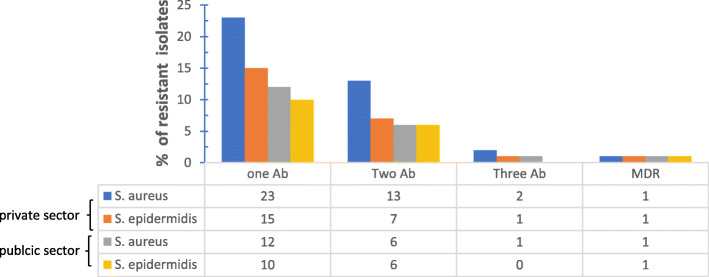
Percentage of *S. aureus* and *S. epidermidis* resistance to single and multiple antibiotics in private and public sectors. Where Ab: antibiotic, MDR: multi-drug resistance

Resistance to antibiotics varied between isolated strains. It ranged from resistance to one antibiotic to resistance to several antibiotics having different modes of action (Fig. 3). Of the 91 isolates of both *S. epidermidis* and *S. aureus,* antibiotic resistance was more prevalent in isolates from private clinic patients (Fig. 6).

## Discussion

This study aimed to assess the prevalence of antibiotic resistant *C. acnes* among a sample of Jordanian patients. While other studies showed that severe acne is more common in the second and third decade and may persist into their sixth decade [4, 5, 19], 62.5% of severe pustular acne was seen between the age of 15–20 in our cohort. This might be related to hormonal changes as well as stresses that this age group undergoes due to high school/college entry exams [20, 21], a well-known stressful period for this age group in Jordan. Moreover, the decreasing severity of acne with age is attributed to the reduction in sebaceous gland activity compared to puberty [22]. Incidence of acne in middle-aged and older adults (above 35 yrs.) was minimal with the highest incidence being at age range between 15 and 35 yrs. (Fig. 2). This pattern of acne distribution was also demonstrated by others [12, 16].

*C. acnes* was isolated from 100 samples with a recovery rate of 86.9%. Staphylococcus species, including *S. epidermidis* and *S. aureus* were isolated from the skin of patients harboring *C. acnes* (Table 1). The presence of *Staphylococcus aureus* as a transient normal flora of the skin is well documented with high carrier rates in the population [15, 23].

*S. epidermidis* has been reported to aggravate inflammatory acne and an imbalance of skin commensals in favor of *S. epidermidis* could aggravate inflammation of the sebaceous unit as a consequence [16]. Claudel et al have recently reported a potential beneficial role of *S. epidermidis* arguing that acne results from a disturbed equilibrium between *C. acnes* and *S. epidermidis* in pilosebaceous units of acne [24]. *S. epidermidis* controls the proliferation of *C. acnes* via the release of succinic acid, a fatty acid fermentation product, which inhibits surface Toll-like receptors of keratinocytes and tumor necrosis factor and suppresses *C. acnes*-induced IL-6 [24, 25].

Although *C. acnes* are known to be very sensitive to a wide range of antibiotic classes, including clindamycin, erythromycin, and cyclines, the percentage of acne patients carrying *C. acnes* strains resistant to these antibiotics is increasing worldwide and differ from one region to another [26–28]. In this study all 100 patients harbored *C. acnes* with varying degrees of resistance to variable antibiotics. Resistance to erythromycin and clindamycin was the highest while isolates were relatively susceptible to tetracycline (Table 2). These results are consistent with the overall resistance pattern throughout the world [12, 28, 29].

Antibiotic resistance patterns varied between isolates. Some *C. acnes* isolates demonstrated resistance to one antibiotic, others to two different antibiotics and yet a third group demonstrated multi-drug resistance towards three different antibiotics (Fig. 3). Multi-drug resistant *C. acnes* was also reported recently by Zhang et al who isolated strains that were simultaneously resistant to clindamycin, erythromycin and moxifloxacin [6].

Similarly, this pattern was also found in the staphylococcal strains isolated from the skin of patients and not from the acne lesions themselves (Fig. 3). Antibiotic resistance patterns between isolates from the same patient have shown similarity (Table 3). Multiple resistances often included resistance towards erythromycin and clindamycin. Cross-resistance between erythromycin and clindamycin is thought to be due to the presence of the erm(X) resistance gene that confers resistance to Macrolide, Lincosamide and Streptogramin B (MLS) group of antibiotics [30]. This explains the similarity in antibiotic resistance patterns between isolates from the same patient suggesting gene- transfer between species*. S. epidermidis* ability to transfer genes via plasmids to other more pathogenic staphylococcal species has been documented [3].

Most *C. acnes* isolates were resistant to the major five antibiotics (erythromycin, clindamycin, doxycycline, trimethoprim / sulfamethoxazole, and tetracycline). However, 97 and 85% of isolates were susceptible to minocycline and levofloxacin respectively (Table 2). Minocycline and levofloxacin are not used frequently in Jordan due to cost. Minocycline’s side effect profile also includes irreversible slate-grey hyper pigmentation of the skin and the development of lupus-like syndrome which both make it a less commonly used agent for the treatment of acne [31]. The high susceptibility rates towards minocycline and levofloxacin accompanied with high resistance rates towards other antibiotics highlights the excessive use/abuse of the latter antibiotics. The high resistance rates towards erythromycin, clindamycin and doxycycline are similar to previously published reports and mirror the antibiotic-use history for the studied patients (Fig. 5). The presence of such resistant strains could explain the clinical unresponsiveness of these patients as well as the high relapse frequency.

It has been previously demonstrated that the use of topical antibiotics in acne results in antibiotic resistance if used for long periods (more than 8 weeks) [5, 32, 33]. Similarly, 21% of *C. acnes* resistance to clindamycin and erythromycin in our study was detected in isolates from patients who used topical antibiotics (results not shown).

In contrast to the majority of patients visiting the public dermatology clinics, patients attending private clinic were previously treated with antibiotics for acne management. This is reflected in the distribution of resistant *C. acnes* isolate. Private clinic patients showed a higher percentage of resistant *C. acnes* than public clinic patients (Fig. 4). This pattern of distribution of resistant strains was not restricted to *C. acnes* but extended to *S. epidermidis* and *S. aureus* (Fig. 6). Thereby, we could correlate prior consumption of antibiotics with higher resistance patterns observed amongst private sector patients. Significant correlation was found between previous antibiotic administration with the prevalence of C*. acnes* resistant strains [34].

Thirty-nine percent of the patients in this study did not receive prior antibiotic treatment, while (61%) of patients received prior antibiotic therapy with mean treatment duration of 6 ± 2 weeks (Fig. 5). All non-previously treated patients carried antibiotic resistant strains, similarly to previously treated ones, indicating that antibiotic resistant strains are circulating and are emerging not only from acne treatment but also as a result of antibiotic use in treatment of other diseases.

The presence of antibiotic resistance in the normal flora of outpatients highlights the possibility of community spread of antibiotic resistant strains through horizontal transfer. In accordance with previous studies, the relationship between resistance and history of antibiotic use remains debated and resistance to antibiotics could be mainly acquired due to contact-mediated spread of resistant *C. acnes* [6, 35].

Comparing the resistance status of *C. acnes* in Jordan to that in other countries; resistance in Jordan was above average for erythromycin, clindamycin, and doxycycline, and almost similar in the case of minocycline. Such results indicate that the frequent use of certain antibiotics has led to the emergence of resistant strains in comparison to minocycline that is not used as much. Outside of acne, erythromycin and clindamycin are commonly used antibiotics. Erythromycin is used in the treatment of upper respiratory tract infections caused by *M. pneumoniae, Legionella* species, beta-hemolytic streptococcus, and *Streptococcus pneumoniae*. Clindamycin is bactericidal to most non enterococcal gram-positive aerobic bacteria and many anaerobic microorganisms and is considered an excellent alternative to beta-lactam antibiotics for treatment of many staphylococcal infection [36]. The co-existence of *C .acnes* resistant strains with a similar resistance pattern indicates the likelihood of horizontal transfer of antibiotic resistance genes across different bacterial species as noted earlier.

In conclusion, our study demonstrates that the prevalence of *C. acnes* antibiotic-resistance in Jordan is above average compared to other countries. We showed higher resistance of *C. acnes* isolates to erythromycin and clindamycin and intermediate resistance to tetracycline, doxycycline, and trimethoprim/sulfamethoxazole. Furthermore, we demonstrated the presence of multi-drug resistant *C.acnes* strains in outpatients in Jordan. Levofloxacin and minocycline demonstrated the best in-vitro activity against the *C. acnes* isolates*.* Finally, *S. aureus* and *S. epidermidis* was also isolated from acne patients and demonstrated a similar antibiotic resistance pattern to *C. acnes*.

## Supplementary information

**Additional file 1.**

## Data Availability

The datasets used and/or analyzed during the current study are available from the corresponding author on reasonable request.

## Abbreviations

Ab: Antibiotic
DA: Clindamycin
CLSI: Clinical and Laboratory Standards Institute
*C. acnes*: *Cutibacterium acnes*
DO: Doxycycline
E: Erythromycin
LEV: Levofloxacin
MLS: Macrolide, Lincosamide and Streptogramin B
MH: Minocycline
MDR: Multi drug resistance
*S. aureus*: *Staphylococcus aureus*
*S. epidermidis*: *Staphylococcus epidermidis*
TE: Tetracycline
SXT: Trimethoprim/sulfamethoxazole
