# Supplementary material for: Antibiotic resistant Cutibacterium acnes among acne patients in Jordan: a cross sectional study
Source: BMC Dermatol. 2020 Nov 17;20:17. doi: 10.1186/s12895-020-00108-9 (PMC7673087; doi:10.1186/s12895-020-00108-9)
Supplement: Supplementary file 1 — Additional file 1. [file 12895_2020_108_MOESM1_ESM.docx]

**(Participants' Consent Form in English)**

| I volunteer to participate in a research study conducted by [Eman Abdelfattah Alkhawaja] from Petra University. I understand that the study is designed to gather information about acne.   1. My participation in this study is voluntary. I understand that I will not be paid for participation. I may withdraw and discontinue participation at any time without penalty. 2. I understand that the researcher will not identify me by name in any reports using information obtained from this questioner, and my confidentiality as a participant in this study will remain secure. Subsequent uses of data will be subject to standard data use policies which protect the anonymity of individuals and institutions. 3. I have read and understood the explanation provided to me. I have had all my questions answered to my satisfaction, and I voluntarily agree to participate in this study. 4. I have been given a copy of this consent form   Name: ………………………………………………………………………..  Address: ……………………………………………………………………..  Telephone Number: ……………………………………………………  Date: …………………………………………………………………………..  Signature of participant: ……………………………………………. |
| --- |

**Questionnaire for Prevalence of antibiotic resistant P. acne among Acne patients in Amman-Jordan:**

**I am a Master student at the University of PETRA faculty of pharmacy. My research project is on the spread of antibiotic resistance in ACNE bacteria. Your assistance is greatly appreciated.**

| **Section I : Demographics** |
| --- |
| 1. sample number |
| 1. Age(years) 15- 20 20-35 36-50 ≥51 |
| 1. Gender Male Female |
| 1. For female ptiant is she using contaceptive medication Yes No |
| 1. Area of sampling face neck back |
| **Section 2 : Acne severity** |
| **1. Type and severity* of acne :**   \| **Severity** \| **Papules/pustules** \| **Nodules** \| \| --- \| --- \| --- \| \| **1. Mild**  **2. Moderate**  **3. Severe** \|  \|  \| |
| **2.Disease duration weeks 3-6 month 6-9 month year > year**   \| Acne classifications* \| \| \| \| \| \| \| --- \| --- \| --- \| --- \| --- \| --- \| \| **Severity** \| \| **Papules/pustules** \| **Nodules** \| \|  \| \|  \| \| \| \| \| \| \| Mild \| Few to several \| \| None \|  \| \| \| Moderate \| Several too many \| \| Few to several \|  \| \| \| Severe \| Numerous and/or extensive \| \| Many \|  \| \| |
| **Section II : Source of Sample:** |
| \| A. First time treated for acne \| \| --- \|  \| **B. previously treated** \| \| --- \| \| **1. Took antibiotic for a week and there's no improvement.**  **2. Took a complete course of anti biotic and improved.**  **3. No improvement and switch to another antibiotic.**  **What was the antibiotic………………………………………………..**  **What is the new antibiotic................................................................** \| \| **C. On antibiotic treatment** \| |
| **Section lll: History of antibiotics** |
| 1. **Has the patient been on antibiotics during the past year ?**   **Yes:**  Oral antibiotic for any reason (what?)  Oral antibiotic for acne  Topical antibiotic  Simultaneous topical oral antibiotic  **No**   1. **For how long did the patient use the antibiotic?**   0-2 weeks  2-6 weeks  6-8 weeks  2-6 month  ≥6 month   1. **Since when did the patient stop using those antibiotics?**   Current use (up to 1 month)  1-4 months  > 4 months  **4. If the patient is using antibiotics for treating acne what is it?**  **Oral Antibiotic**  Doxycycline and Minocycline  Tetracycline  Erythromycin  Clindamycin  Combination of two antibiotics …………………………  **Topical antibiotic:**  Clindamycin  Erythromycin |
